# A Rare Presentation of Plexiform Schwannoma of the Thumb

**DOI:** 10.1016/j.jhsg.2023.01.005

**Published:** 2023-04-03

**Authors:** Roxana Martinez, Akinola Emmanuel Oladimeji, Robert H. Wilson

**Affiliations:** ∗ Department of Orthopaedic Surgery, Howard University Hospital, Washington, DC

**Keywords:** Plexiform schwannoma, Schwannoma, Thumb mass

## Abstract

Plexiform schwannomas are rare, benign, neural crest-derived tumors that commonly occur in the hand and upper extremities. They may be sporadic or associated with neurofibromatosis type 2. Although previous literature has described plexiform schwannomas occurring in fingers, nerve and tendon sheaths, and intraosseous lesions, this is the first known case of a plexiform schwannoma of the thumb. This is a case of a growing, painless, subungual mass of the thumb in a 54-year-old patient. After surgical excision and subsequent immunohistochemical examination, the patient was diagnosed with a plexiform schwannoma. This highlights the importance of maintenance a broad differential before surgery and obtaining a proper diagnosis using histopathology.

Schwannomas, also known as neurilemmomas, are benign, neural crest-derived proliferations of peripheral nerve sheath cells. These masses account for approximately 5% of all tumors involved with the upper extremities and 2.8% of biopsied benign, soft-tissue tumors of the hand and wrist.^1^ These lesions typically occur along peripheral nerves and/or along the volar aspect of digits, hands, or arms. They may be associated with neurofibromatosis type 2, although they may also occur sporadically. The histologic variants of schwannomas include cellular, melanotic, and plexiform subtypes. Plexiform schwannomas account for approximately 5% of all schwannomas.^2^ They are given their name based on of their growth pattern, which distorts the affected nerve section, giving it a multivermiform appearance.

This report describes a rare case of a plexiform schwannoma of the volar aspect of the thumb in a 54-year-old patient. After surgical excision and subsequent immunohistochemical examination, the patient was diagnosed with a plexiform schwannoma. Written informed consent was obtained from the patient for publication of this case report and accompanying images.

## Case Report

**Figure 1:**
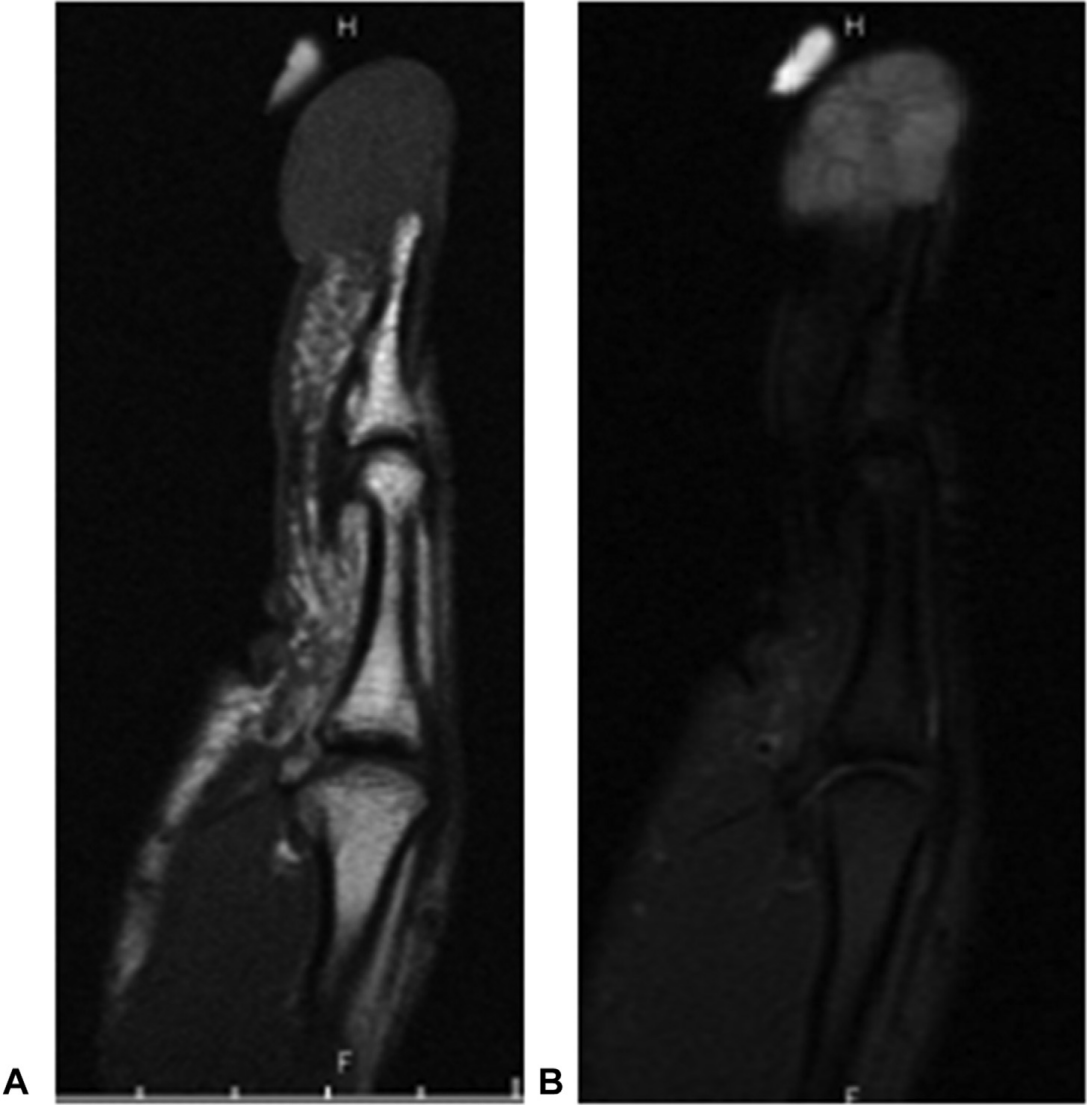
T1 and T2 fat-suppressed sagittal imaging of the right thumb. These illustrated a **A** T1-hypointense and **B** T2-hyperintense loculated mass volar and distal to distal phalanx. There were no signs of cortical disruption or boney lesions.

A 54-year-old woman with a medical history of chronic kidney disease stage 2 and prediabetes mellitus presented for the evaluation of a solitary, painless mass over the subungual region of her right thumb. The mass had been growing for approximately 7 years but doubled in size over the past year, resulting in a notable deformity. Because the patient was dissatisfied with the cosmetic appearance of her thumb, she requested surgical excision. Radiographs were obtained and magnetic resonance imaging was performed to assess for scalloping of the distal phalanx. The radiographs of the thumb were unremarkable. Magnetic resonance imaging showed a septated, large volar mass along the distal phalanx with low intensity on T1 and high intensity on fluid sensitive sequences (Fig. 1). After going through the potential risks and benefits of surgery, informed consent was obtained for excision of the mass of the right thumb.

**Figure 2:**
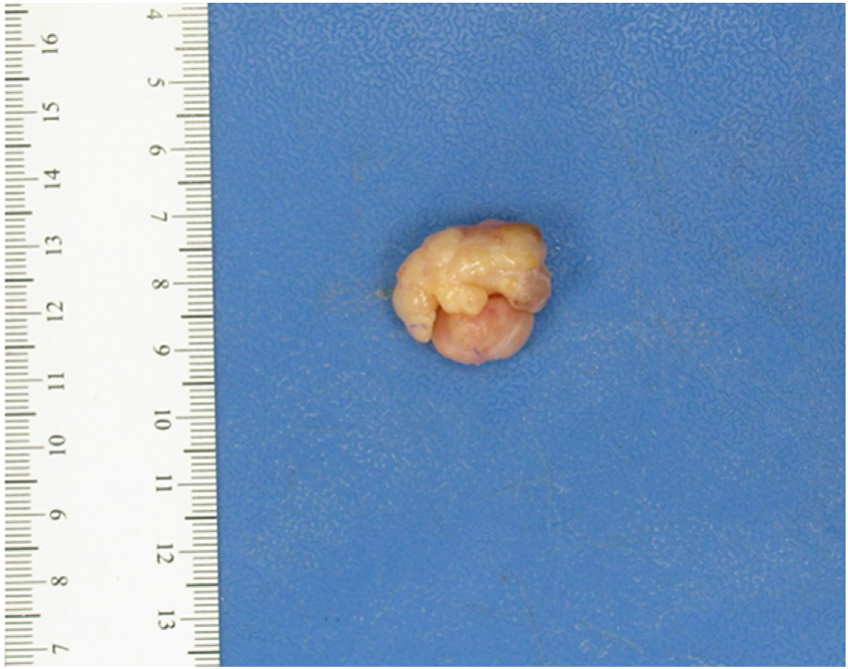
Gross specimen demonstrating a well-circumscribed, lobulated, gray or white and yellow, firm-to-soft tissue measuring 2.5 × 2.3 × 1.5 cm^3^.

The patient was brought to the operating room and placed in a supine position. After the patient was placed under general anesthesia, the right upper extremity was prepared and draped in the usual sterile fashion. The upper extremity was exsanguinated using an Esmarch bandage (Cardinal Health). The patient had a prosthetic nail in place, which was trimmed back in order to gain access to the hyponychium. A fishmouth-style incision was made around the tip of the thumb, after which the skin over the lesion was peeled back. The lesion appeared to be soft and well circumscribed. It was gray and white in color and approximately 2 cm in diameter. The entire mass was excised in 1 piece and sent to the pathology department for review (Fig. 2). Under loupe magnification, any remaining tissue that appeared to be consistent with the mass was debrided. The soft tissue of the pulp was displaced proximally, after which excess skin was excised. The tourniquet was released, hemostasis was achieved, and the incision was closed using interrupted absorbable sutures. A digital block was performed, and the digit was placed in a protective splint. The patient was awakened and transferred to the recovery room in a stable condition.

**Figure 3:**
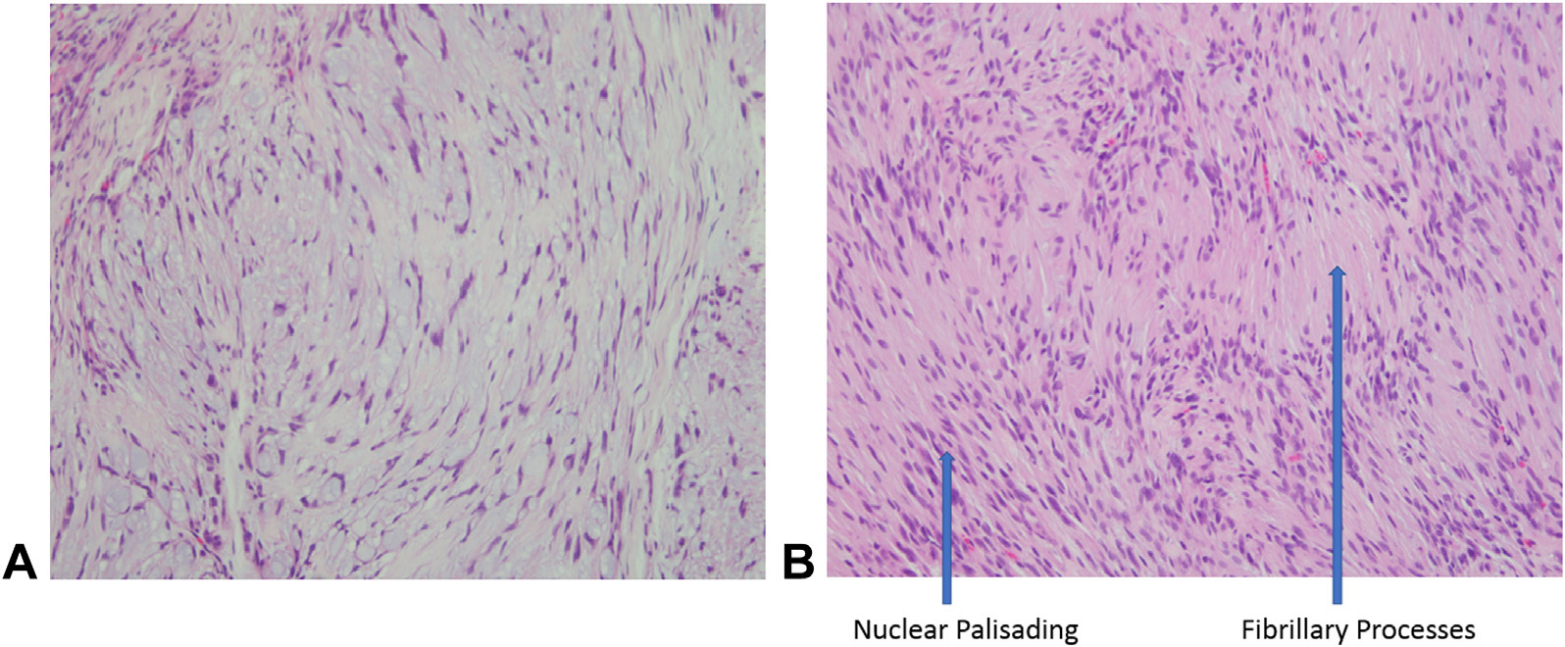
**A** A high-power view showing hypocellular areas with a myxoid background. **B** Hypercellular areas showing nuclear palisading around fibrillary processes (verocay bodies). The tumor cells had wavy, slender nuclei with tapering ends, indicating neural differentiation. There was no atypia, mitoses, or necrosis, ruling out malignancy.

**Figure 4:**
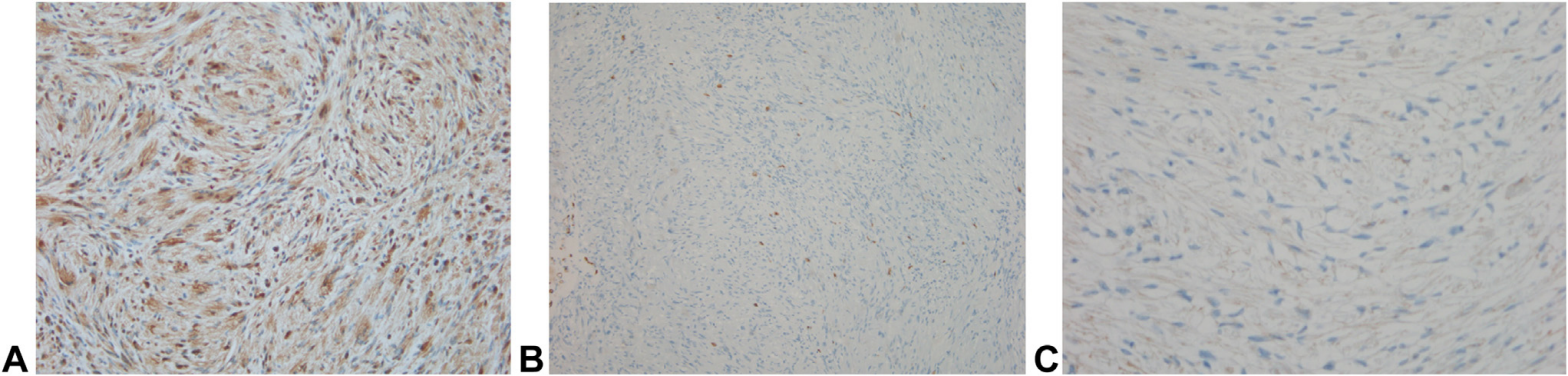
**A** S-100 immunohistochemical (IHC) staining showed variable nuclear and cytoplasmic positivity. **B** The CD34 IHC staining result was negative, ruling out neurofibroma. **C** Epithelial membrane antigen IHC staining highlighted the peripheral perineural cells but showed only occasional staining in the central proliferating tumor cells. This pattern and the diffuse S-100 positivity ruled out perineurioma.

The surgical pathology department examined the excised specimen and identified a well-circumscribed tumor with a myxoid background and foci of microcytic changes as well as wavy, slender nuclei with tapering ends, suggestive of neural differentiation (Fig. 3). The tumor stained positive for S-100 and negative for CD34 (Fig. 4). Epithelial membrane antigen staining showed occasional centrally proliferating tumor cell staining and peripheral perineural staining. The overall histologic and immunochemical findings were most suggestive of plexiform schwannoma.

**Figure 5:**
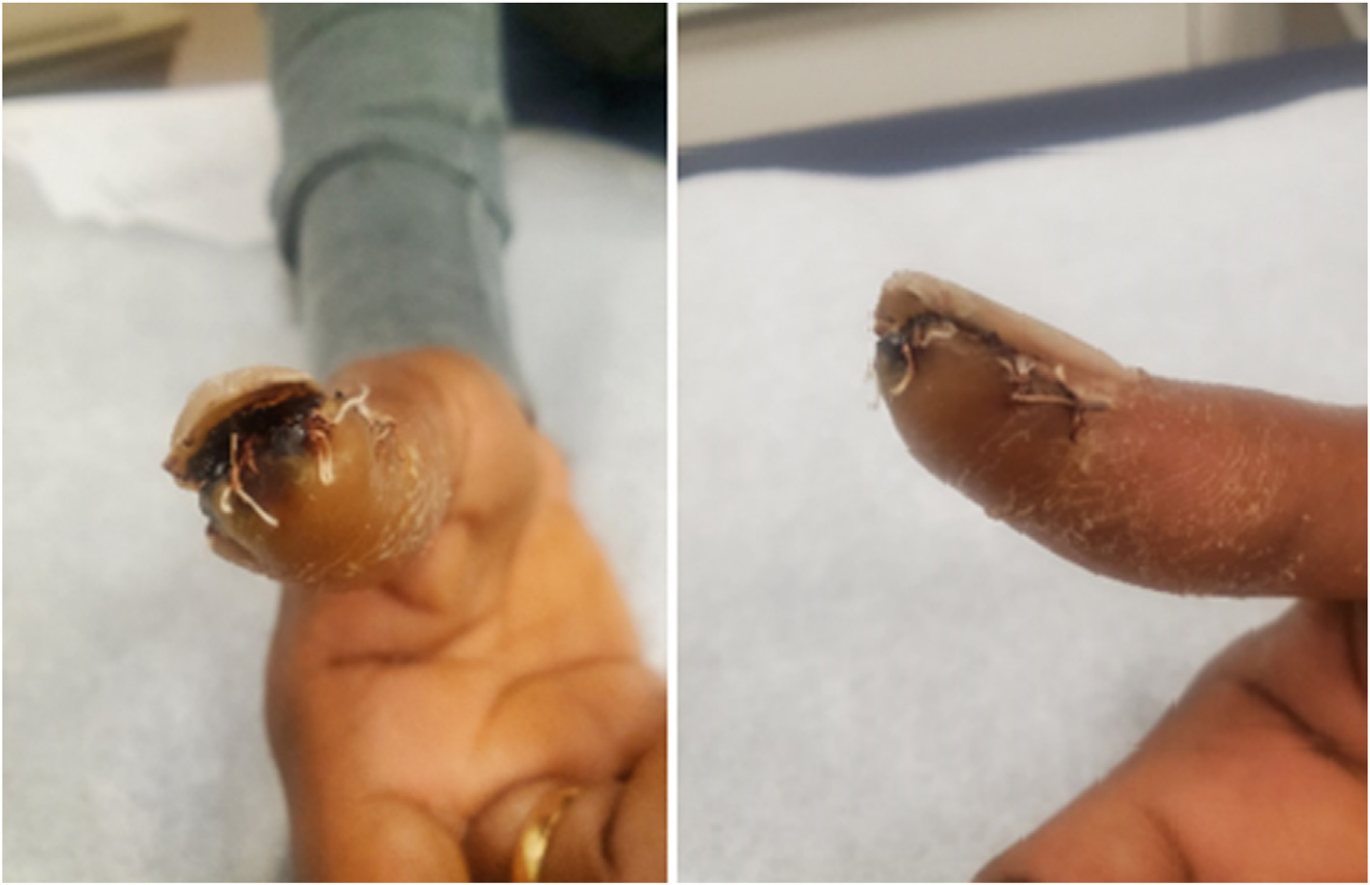
A clinical photograph 1 month after the surgery demonstrating a well-healing incision.

**Figure 6:**
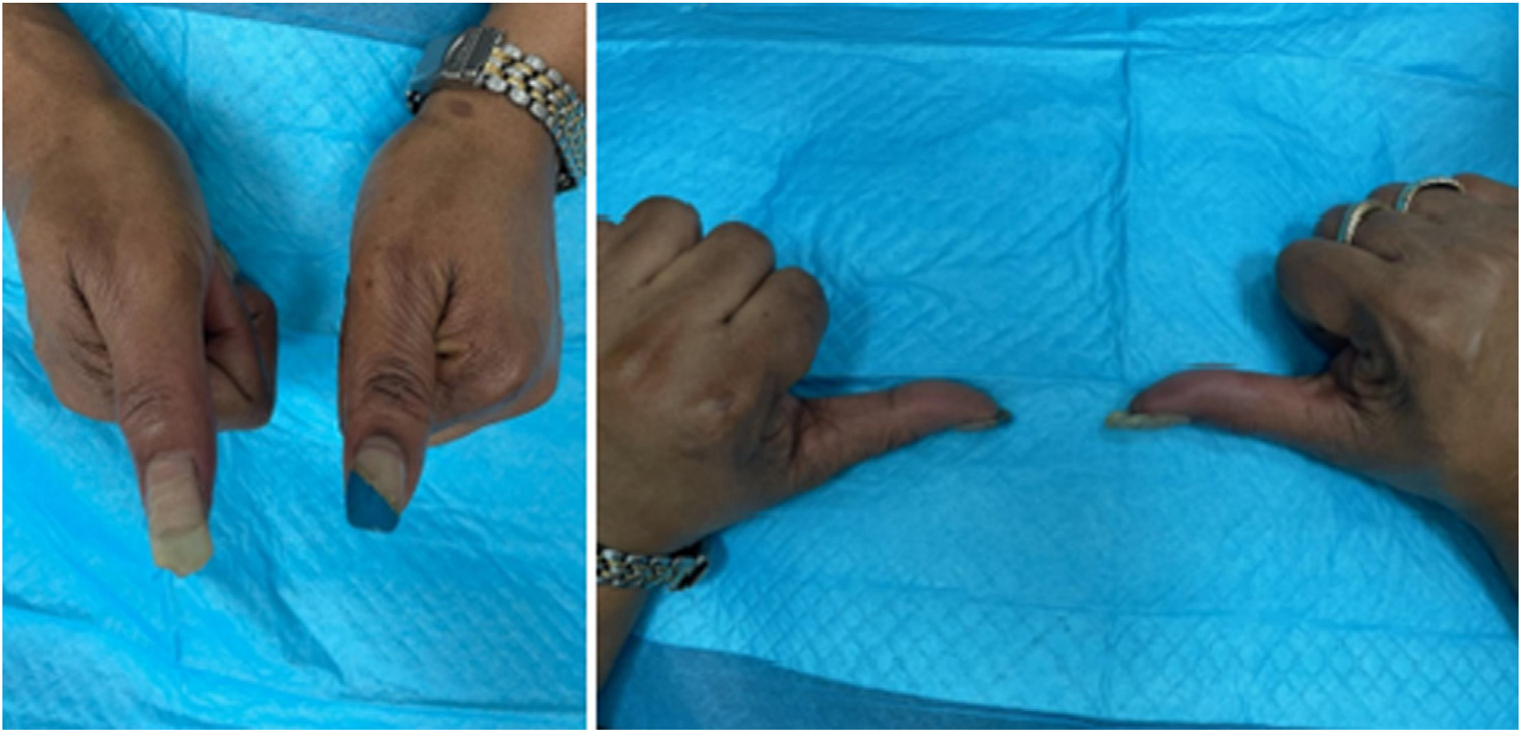
A clinical photograph 2 months after the surgery demonstrating a well-healed scar.

After surgery, the patient’s incision healed well and the thumb remained neurovascularly intact (Figs. 5, 6). She was satisfied with her outcomes after the surgery. At her most recent visit, 6 months after the surgery, she had a new complaint of a soft, painless, enlarging mass of the thigh. She was referred to a lower-extremity specialist for further evaluation.

## Discussion

Plexiform schwannomas are solitary, benign, peripheral nerve sheath tumors that typically localize in nerve plexuses, the skin, and subcutaneous tissues of the upper and lower extremities.^3^ The other locations where these tumors have been observed include the little finger and, intraosseously and most commonly, the mandible, with no associated peripheral nerves.^4^^,^^5^ Some other unusual cases have also been associated with patients exhibiting macrodactyly and trigger finger.^6^^,^^7^ To the best of our knowledge, this is the first reported case of a plexiform schwannoma occurring in a thumb.

Plexiform schwannomas are not visible on radiographs but have a hyperintense signal on T2 on magnetic resonance imaging, as seen in this case.^1^ Many plexiform schwannomas observed in a patient at once may be classified as schwannomatosis; there is a higher association of schwannomatosis with neurofibromatosis type 2 than of solitary masses.^2^ Although plexiform schwannomas have no mitoses and lack the potential for malignant transformation, pleomorphic nuclei are commonly seen on histologic investigation, although this was not seen here. These tumors may be characterized by a biphasic pattern of cell organization: Antoni type A and Antoni type B. Antoni type A consists of proliferating spindle-shaped cells tightly packed into bundles with foci of nuclear palisading and verocay bodies, whereas Antoni type B is hypocellular and consists of lipid-containing cells organized into myxoid stroma (Fig. 3).^1^

The immunohistochemical markers used to detect plexiform schwannomas include S-100 (a marker for cells originating from the neural crest), calretinin or CD56, SMA, and desmin.^2^^,^^8^ These markers are important for differentiating between plexiform neurofibromas and plexiform schwannomas.^2^ For instance, plexiform schwannomas are exclusively composed of Schwann cells, whereas plexiform neurofibromas consist of a combination of disorganized arrays of Schwann cells, fibroblasts, and axons, with a prominent myxoid matrix, which is not observed in plexiform schwannomas.^9^ Plexiform neurofibromas are weakly S-100+ with focal staining, and there is neurofilament activity within the tumors’ nodules, as seen in this case. The options for treatment include simple observation because of its very rare potential for malignant transformation or surgical excision, usually for cosmetic purposes or pain relief.

This case was unusual given the location of the plexiform schwannoma in the thumb. This highlights the importance of maintenance of a broad differential before surgery and obtaining a proper diagnosis using histopathology. The diagnosis of 1 lesion may help guide management for future lesions found later (eg, the mass in this patient’s leg). Ultimately, histologic assessment is required for definitive diagnosis of plexiform schwannomas.
